# Detection of Resting-State Functional Connectivity from High-Density Electroencephalography Data: Impact of Head Modeling Strategies

**DOI:** 10.3390/brainsci11060741

**Published:** 2021-06-03

**Authors:** Gaia Amaranta Taberna, Jessica Samogin, Marco Marino, Dante Mantini

**Affiliations:** 1 Research Center for Motor Control and Neuroplasticity, KU Leuven, 3001 Leuven, Belgium; gaia.taberna@kuleuven.be (G.A.T.); jessica.samogin@kuleuven.be (J.S.); marco.marino@kuleuven.be (M.M.); 2 Brain Imaging and Neural Dynamics Research Group, IRCCS San Camillo Hospital, 30126 Venice, Italy

**Keywords:** electroencephalography, functional connectivity, resting-state networks, head modelling, electrode localization, head tissue segmentation

## Abstract

Recent technological advances have been permitted to use high-density electroencephalography (hdEEG) for the estimation of functional connectivity and the mapping of resting-state networks (RSNs). The reliable estimate of activity and connectivity from hdEEG data relies on the creation of an accurate head model, defining how neural currents propagate from the cortex to the sensors placed over the scalp. To the best of our knowledge, no study has been conducted yet to systematically test to what extent head modeling accuracy impacts on EEG-RSN reconstruction. To address this question, we used 256-channel hdEEG data collected in a group of young healthy participants at rest. We first estimated functional connectivity in EEG-RSNs by means of band-limited power envelope correlations, using neural activity estimated with an optimized analysis workflow. Then, we defined a series of head models with different levels of complexity, specifically testing the effect of different electrode positioning techniques and head tissue segmentation methods. We observed that robust EEG-RSNs can be obtained using a realistic head model, and that inaccuracies due to head tissue segmentation impact on RSN reconstruction more than those due to electrode positioning. Additionally, we found that EEG-RSN robustness to head model variations had space and frequency specificity. Overall, our results may contribute to defining a benchmark for assessing the reliability of hdEEG functional connectivity measures.

## 1. Introduction

Electroencephalography (EEG) is a non-invasive neurophysiological technique that measures the variation in electrical potentials by means of electrodes positioned over the scalp [1]. This variation in potentials is associated with neural activity that is generated inside the brain, referred to as source space, and propagates to the surface, named sensor space [1]. The analysis of EEG recordings can be used as a proxy to better understand neural mechanisms in health and disease. So far, most EEG studies have relied on analyses conducted in the sensor space, to make inferences on neural responses to specific stimuli (event-related potential analysis) [2,3] or modulations of neural oscillations during task performance (event-related synchronization/desynchronization analysis) [4,5,6,7].

Over the last years, there has been a growing interest in using electrophysiological techniques to study functional interactions between distant brain regions, namely functional connectivity, especially in the resting-state condition. Different resting-state networks (RSNs), among which are the default mode (DMN), the dorsal (DAN) and the ventral (VAN) attention, the language (LN), the somatomotor (SMN) and the visual (VN) networks, have been detected first using magnetoencephalography [8,9] and then EEG [10,11,12,13]. The use of high-density EEG (hdEEG) systems capable of recording more than 100 signals at different scalp locations have been crucial to reliably measure functional connectivity and map RSNs [14,15,16,17]. Specifically, dedicated analysis workflows for hdEEG data were developed to improve the reconstruction of neural activity in the brain [10,11,12,18,19,20,21], including tools for signal pre-processing, head modeling and source localization. The use of an appropriate head modeling strategy is deemed fundamental to take advantage of the superior scalp coverage provided by hdEEG [22,23,24,25]. The localization of neural generators requires indeed the modeling of neural activity propagation from the sources, located inside the brain, to the potentials measured by EEG sensors over the scalp. The linear relationship between source activity and EEG measures is mathematically modeled by means of the leadfield matrix [17,26]. Notably, an accurate realistic head model has been found to have a direct positive impact on the performance of source localization algorithms [14,16,17,27,28,29]. Two factors primarily contribute to the accuracy of head modeling: head image segmentation and EEG electrode positioning.

The segmentation of the head image, which can be obtained using the magnetic resonance (MR) imaging technique, permits the modeling of the topological and physical properties of each head tissue compartment. To this end, different MR segmentation methods were proposed, whose accuracy depends on the space where such segmentation is performed—individual [22,25,30] or template [31,32,33,34]—and on the number of considered tissues, which typically ranges from 3 to 12 [10,22,25,30,35,36,37,38]. In a recent study, we introduced MR-TIM, a computational method for automated head image segmentation in 12 tissue classes [25]. Importantly, we demonstrated the superior performance of MR-TIM compared to existing methods, which can identify 12 tissue classes by warping a segmented template image to individual space [10], or can segment the real MR image into 3 to 5 tissue classes directly in the individual space [35,38].

The accuracy of electrode positions is also very important for the generation of the leadfield matrix. Electrode positions can be obtained before or after the EEG measurements using digitization [39,40], photogrammetry [41,42] and 3D scanning [43,44,45,46] techniques. These are influenced to a variable extent by single-electrode localization errors [47] as well as by errors related to the co-registration of the whole set of electrodes to the head shape [48]. To the best of our knowledge, no study has yet assessed the impact of different electrode positioning, as well as of head tissue segmentation techniques, on functional connectivity measured on source-reconstructed hdEEG data.

In this study, we used real hdEEG data to examine the RSN reconstruction performance that can be achieved thanks to an optimized version of our hdEEG analysis workflow [49], including newly-developed methods for realistic MR segmentation in 12 head tissue compartments [25] and for leadfield matrix generation by finite difference modeling [50]. The connectivity patterns that we obtained were used as a reference to assess to what extent EEG-RSNs are sensitive to head model inaccuracies. We first tested the impact of random and systematic errors in electrode positioning, and—from this analysis—we derived the potential reduction in RSN reconstruction performance associated with the use of 3D scanning and digitization techniques for electrode localization. We then extended our performance analysis to different head tissue segmentations based on MR data (with twelve or with three tissues, either based on the individual MR image or on a template image). This allowed us to evaluate whether electrode positioning or head tissue segmentation most strongly influences RSN reconstructions from hdEEG data. Finally, we assessed if all RSNs are influenced in a similar manner by the methods used for electrode positioning and head tissue segmentation, or if—alternatively—some RSNs are less robust than others.

## 2. Materials and Methods

### 2.1. EEG Experiment

Eyes-open resting-state data were collected in a cohort of 19 healthy young adults (age 28 ± 6 years, 5 males) and were previously used in other studies from our group [11,12,49]. Ethical approval was granted by the Ethics Committee of ETH Zurich; informed consent was obtained from all participants and the experiment was performed in accordance with the relevant guidelines and regulations. For each participant, we recorded hdEEG signals for 5 min at 1000 Hz sampling rate using a 256-channel HydroCel Geodesic Sensor Net by Electrical Geodesics (Eugene, OR, USA). Electromyograms and vertical and horizontal electrooculograms (EOG) were collected in addition to the hdEEG signals. Positions of the EEG sensors and of three landmarks (nasion, left and right preauricular points) were localized using a Geodesic Photogrammetry System (GPS) [42]. In a separate session, we acquired a T1-weighted whole-head anatomical image using a Philips Ingenia 3T MR scanner (Best, The Netherlands) with a turbo field echo sequence. Scanning parameters were: TR = 8.25 ms, TE = 3.8 ms, flip angle = 8°, voxel size = 1 mm^3^ isotropic.

### 2.2. Standard EEG Data Analysis

Functional connectivity was estimated from hdEEG data using an automated analysis workflow developed in the MATLAB^®^ (MathWorks Inc., Natick, MA, USA) environment. The workflow consisted of four main steps: pre-processing of EEG signals; creation of individual head model; reconstruction of cortical activity; and seed-based connectivity analysis. Each step is briefly described in the following sections; for a more detailed explanation refer to [10,12,49].

#### 2.2.1. EEG Signal Pre-Processing

In order to clean EEG recordings from noise and biological artefacts, we first identified channels with poor signal quality and corrected them by spatially interpolating the signals from their neighbors, as defined using the FieldTrip toolbox (http://www.fieldtriptoolbox.org/, accessed on 2 July 2019). We band-pass filtered the data in 1–80 Hz and then removed EOG, muscular, movement and biological artefacts using Independent Component Analysis (ICA). Independent components that were not classified as artefactual were linearly mixed to reconstruct artefact-free channel data [51]. Finally, the pre-processed EEG data were re-referenced using the Reference Electrode Standardization Technique [51,52,53,54].

#### 2.2.2. Realistic Head Model Creation

An individual head model was reconstructed using the MR image of the participant and the EEG sensor positions. The individual MR image was segmented in 12 head compartments (skin; eyes; muscle; fat; spongy bone; compact bone; cortical grey matter; cerebellar grey matter; cortical white matter; cerebellar white matter; cerebrospinal fluid; brain stem) using the MR-TIM software [25]. The conductivity value of each tissue compartment was assigned based on previous literature [55]. Electrode positions were extracted from the GPS data, and projected over the head surface to minimize localization errors. Afterwards, a 3D regular 6 mm grid overlapping with the cortical/subcortical and cerebellar compartments was generated, to define all possible dipolar sources. Finally, a whole-head conductivity model was created using the optimized finite difference method described in [50]. Based on this model, the leadfield matrix, expressing the linear relationship between scalp EEG data and source-space neural activity, was calculated.

#### 2.2.3. Source Activity Reconstruction

The pre-processed EEG signals were then combined with the realistic head model to compute the cortical three-dimensional distribution of current density using the exact low-resolution brain electromagnetic tomography (eLORETA) [56]. The eLORETA method is a weighted minimum norm inverse solution, where the weights are unique and the inverse solution provides exact localization for any point source in the brain. In this way, cortical neural activity was estimated with high-temporal resolution in a 6 mm homogeneous grid constrained to the grey matter.

#### 2.2.4. Functional Connectivity

**Figure 1:**
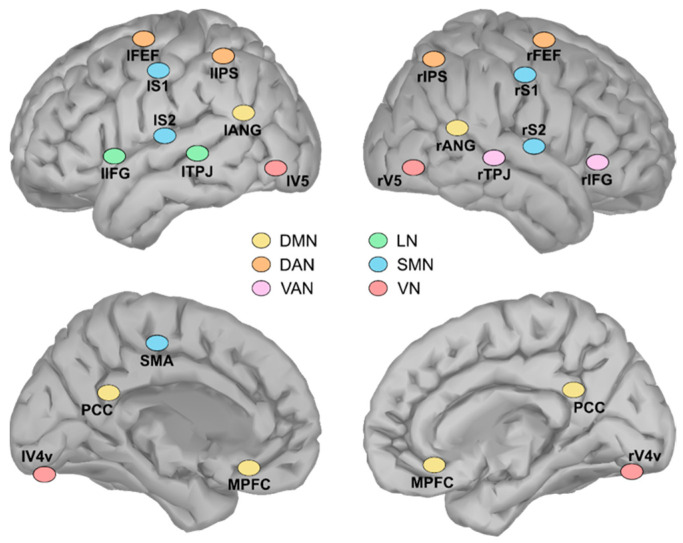
Anatomical positions of the 21 seeds used for analysis, subdivided into the corresponding networks. Default mode network (DMN): posterior cingulate cortex (PCC), medial prefrontal cortex (MPFC), left/right angular gyrus (lANG/rANG); Dorsal attention network (DAN): left/right frontal eye field (lFEF/rFEF), left/right inferior parietal sulcus (lIPS/rIPS); Ventral attention network (VAN): right temporo-parietal junction (rTPJ), right inferior frontal gyrus (rIFG); Language network (LN, green): left temporo-parietal junction (lTPJ), left inferior frontal gyrus (lIFG); Somatomotor network (SMN): supplementary motor area (SMA), left/right primary somatosensory cortex (lS1/rS1), left/right secondary somatosensory cortex (lS2/rS2); Visual network (VN): left/right human ventral visual area 4 (lV4v/rV4v), left/right visual area 5 (lV5/rV5).

We reconstructed functional interactions within and between six large-scale RSNs: DMN, DAN, VAN, LN, SMN and VN. In order to estimate the frequency-specific interactions, we selected a total of 21 nodes within the main regions of the six RSNs (Figure 1 and Table S1), based on previous studies [49,57,58,59,60].

Node coordinates, defined in MNI space, were projected onto the participant’s cortical space. We defined 6 mm radius spherical regions of interest (ROIs) centered over the seed coordinates. Time courses of these seed ROIs were decomposed in the time–frequency domain using the short-time Fourier transform, with Hamming windows of 2 s and a 50% overlap between consecutive windows. Connectivity values were estimated between each pair of seeds using band-limited power envelope correlations. Prior to that, we performed a frequency-by-frequency orthogonalization [61] to remove spurious interactions at zero-lag due to signal leakage. Logarithmic-transformed signal-orthogonalized power time-courses were correlated and resulting correlations were converted to z-values using the Fisher’s transform [61,62]. The average connectivity between the seeds of the same network was defined as the intra-network connectivity. Similarly, the average connectivity between the seeds associated with two different networks was defined as the inter-network connectivity [63,64]. We examined the strength of network interactions corresponding to delta (1–4 Hz), theta (4–8 Hz), alpha (8–13 Hz), beta (13–30 Hz) and gamma (30–80 Hz) bands, by averaging the z-values associated with the frequencies within the relevant range. In this manner, we derived band-limited RSN correlation matrices. Finally, for each network and frequency band separately, we used a one-tailed Wilcoxon signed rank test to contrast intra- and inter-network connectivity values.

### 2.3. Impact of Head Modeling Strategies

Several test models were created, by varying the reference analysis workflow described above with respect to a single parameter at the time. These tests were focused on assessing electrode localization errors and head tissue segmentation accuracy, respectively. For each test model, the corresponding analysis workflow was re-run on the hdEEG data, to produce new connectivity results for: (1) band-limited RSN correlation matrices and (2) the contrast between intra- and inter-network connectivity for each RSN and frequency band. To test the reliability of each model, we calculated Spearman’s correlation values between the connectivity matrices reconstructed from the reference analysis workflow and those obtained from the specific test model. The correlation values were transformed to z-values using the Fisher transformation to improve data normality.

#### Generation of Test Models

**Figure 2:**
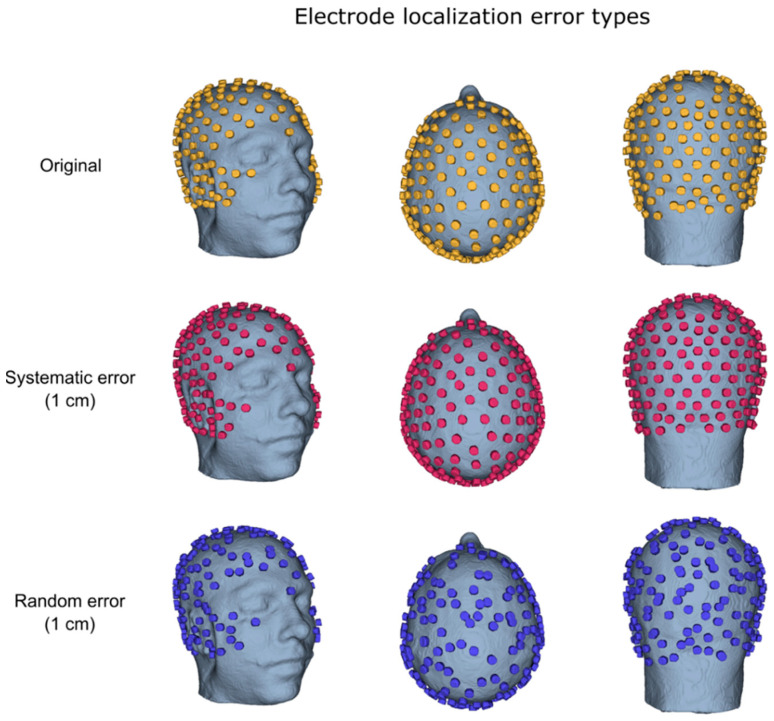
Example of EEG electrode positions and their simulated misplacement. From top to bottom: original electrode positions for a representative participant; positions with a systematic error of 1 cm; positions with a random error of 1 cm.

In order to evaluate the impact of electrode localization errors on the connectivity results, we created eight sets of synthetic electrode positions by adding displacements to the reference electrode positions (Figure 2). These displacements differed in magnitude and type. In particular, we defined six possible rotations around the anteroposterior, the mediolateral and the longitudinal axes of the head. The amplitude of the rotation determined the displacement of each electrode, which was defined according to previous studies [45,48,65], and was set to approximately: 0.25, 0.5, 0.75 and 1 cm. Within each dataset, the same direction of rotation was applied to all the electrodes to simulate the co-registration error (“*Systematic*”), whereas a random rotation was applied to each electrode for the localization error (“*Random*”). Rotations were randomized across datasets.

As a second analysis step, we simulated the impact of electrode position errors that are typical of 3D scanning [43,44,45] and digitization techniques [39,40]. The synthetic set of electrode positions for 3D scanning (*3D Scan*) was created by considering a systematic error equal to 0.25 cm and no random error; for the digitization one (*Digitizer*) we included a systematic error of 0.5 cm and a random error of 0.25 cm [44,45,65] (Table S2). The amplitude of the rotation was constant for all the datasets, while the direction of the rotation was randomized across participants.

**Figure 3:**
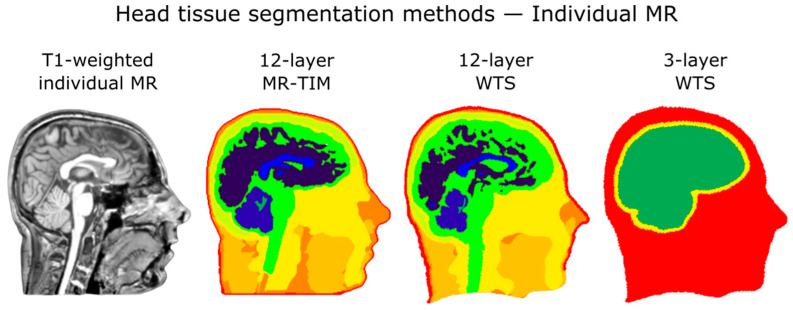
Example of head tissue segmentations used in the study. From left to right: T1-weighted individual MR image; 12-layer segmentation using the MR-TIM toolbox; 12-layer segmentation using WTS; 3-layer segmentation using WTS.

We also examined the impact of the head tissue segmentation on the connectivity results, by using three different segmentation approaches with decreasing levels of complexity (Figure 3). First, we applied the warping of template segmentation (WTS) technique [10,25] on the individual MR image to obtain a “*12-layer WTS*” segmentation, with the same compartments defined by MR-TIM [25]. Then, the “*3-layer template*” segmentation was generated by clustering different compartments of the 12-layer template (brain: cortical and cerebellar white and grey matter, brainstem, cerebrospinal fluid; skull: compacta and spongiosa; scalp: muscle, fat, eyes, skin) [66]. Finally, we used the WTS technique to generate a “*3-layer WTS*” segmentation in individual space, based on the 3-layer template segmentation (Figure S1). For the *12-layer* and *3-layer WTS* we used the reference electrode positions in individual space; conversely, we used template electrode positions of the 256-channel HydroCel Geodesic Sensor Net for the segmented image in template space.

In addition, we created matrices contrasting intra- and inter-network connectivity. To this end, we used a one-tailed Wilcoxon signed rank test for the Spearman’s correlation values derived from *12-layer WTS*, *3-layer WTS*, *3-layer template*, *3D Scan* and *Digitizer* test models. These correlation values were obtained using the corresponding connectivity matrix derived from the standard analysis workflow as reference. With such comparisons, we aimed at understanding how robust the connectivity pattern of each network in each frequency band was when varying either the electrode positioning or the head tissue segmentation technique.

### 2.4. Statistical Analysis

For the analysis of electrode localization errors, we used a four-way analysis of variance (ANOVA) on the z-values to estimate to what extent the following factors influenced the connectivity values: network (DMN; DAN; VAN; LN; SMN; VN), frequency band (delta; theta; alpha; beta; gamma), error magnitude (0.25; 0.5; 0.75; 1 cm) and error type (systematic; random). A multiple comparison test was also performed to investigate the differences among estimated marginal group means, for each significant factor [67]. Furthermore, we compared correlation values corresponding to systematic and random localization errors to investigate in more detail under which conditions the two errors yield significantly different connectivity results. To this purpose, we used a two-tailed Wilcoxon signed rank paired test with significance level set to *p* < 0.05, corrected for multiple comparisons using the Bonferroni method. In order to test the impact of the head tissue segmentation method on connectivity, we performed a three-way ANOVA with factors: network (DMN; DAN; VAN; LN; SMN; VN), frequency band (delta; theta; alpha; beta; gamma) and segmentation method (*12-layer WTS*; *3-layer WTS*; *3-layer template*). Finally, we used a two-tailed Wilcoxon signed-rank paired test to check for differences in connectivity values between the head tissue segmentation and electrode positioning techniques. The significance level was set to *p* < 0.05, corrected for multiple comparisons using the Bonferroni method.

## 3. Results

In this study, we aimed at investigating the impact of the head model accuracy on EEG-RSN connectivity estimation, particularly focusing on the role of different electrode localization techniques and head tissue segmentation methods.

**Figure 4:**
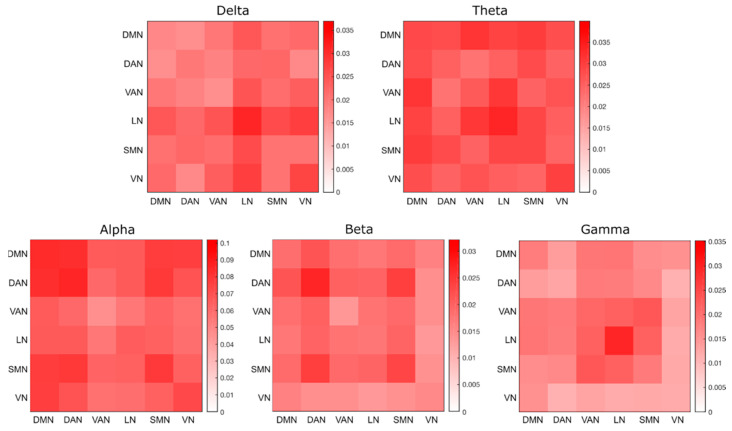
Functional connectivity matrices for the following frequency bands: delta (1–4 Hz), theta (4–8 Hz), alpha (8–13 Hz), beta (13–30 Hz) and gamma (30–80 Hz). In each matrix, the diagonal values represent the group average intra-network connectivity, whereas the off-diagonal values represent the group average inter-network connectivity.

**Figure 5:**
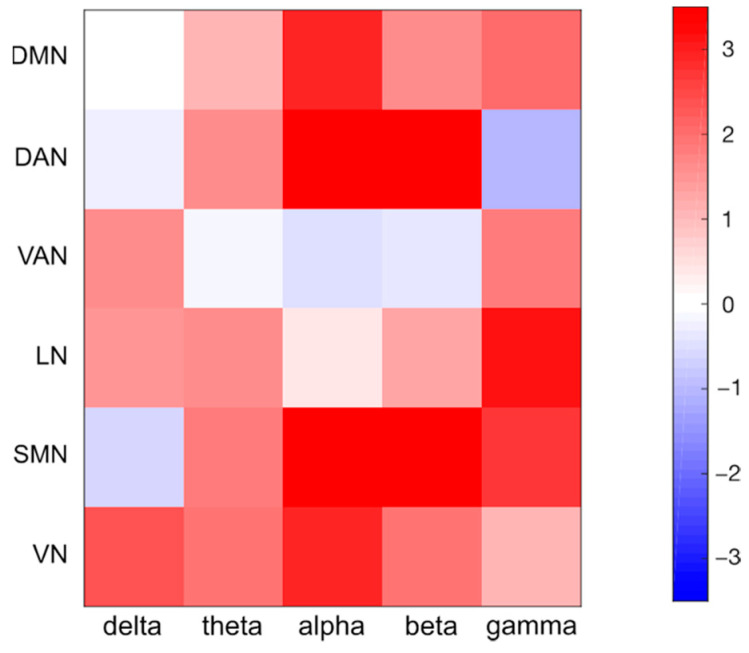
Intra- and inter-network connectivity differences per network and frequency band. A comparison was performed by means of a Wilcoxon signed-rank paired test; the color bar represents the z-value obtained through the test, where the red colors stand for intra-network connectivity values higher than inter-network ones, and the blue colors vice-versa.

First, we reconstructed the functional connectivity values using our reference data. In particular, we quantified the connectivity between pairs of RSNs for each frequency band (Figure 4), and the differences between intra- and inter-network connectivity for each network and frequency band (Figure 5 and Figure S2). We observed remarkable similarities with connectivity results obtained in our recent study [49]. This was a valuable starting point for a detailed analysis of the impact of different head models on RSN connectivity.

**Figure 6:**
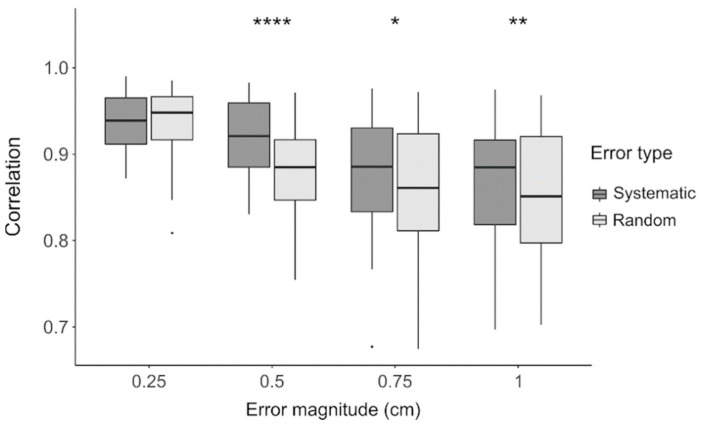
Correlation between the reference connectivity matrices and those obtained when introducing electrode localization errors. For each error, magnitude is tested in the range 0.25 to 1 cm, and distinction is made between systematic (dark grey) and random (light grey) errors. Black dots represent the outliers. The asterisks define the significance level of the two-tailed Wilcoxon signed-rank paired test between error types, for each error magnitude: * for *p* < 0.05, ** for *p* < 0.01, **** for *p* < 0.0001.

The ANOVA performed on the z-values extracted for electrode positioning errors revealed significant modulations of all four factors under investigation (*p* < 0.001 for RSN, frequency band, error magnitude and type) (Table 1). Post-hoc tests showed that DMN, DAN and SMN were the most robust networks, whereas VAN and LN were the most sensitive to electrode positioning accuracy (Figure S3A). Similarly, alpha and delta bands were the most and least robust frequency bands, respectively (Figure S3B). As expected, our analyses confirmed that the difference between reference and test connectivity data increased as the magnitude of the positioning error was larger (Figure S3C). The Wilcoxon signed rank test yielded significantly lower correlations with random errors compared to systematic ones, for a magnitude level larger than 0.25 mm (Figure S3D and Figure 6 and Table S3).

The ANOVA run on the z-values obtained with different head tissue segmentation methods showed significant modulations (*p* < 0.001) of all three factors under investigation: RSN, frequency band and segmentation method (Table 2). Once again, post-hoc tests revealed that DMN, DAN and SMN were more robust in regard to head tissue segmentation variations than VAN and LN (Figure S4A), and that alpha and delta bands were the least and most sensitive frequency bands, respectively (Figure S4B). The *3-layer template* method was found to be the least accurate. Notably, no significant differences were observed between the *12-* and *3-layer WTS* methods (Figure S4C).

**Figure 7:**
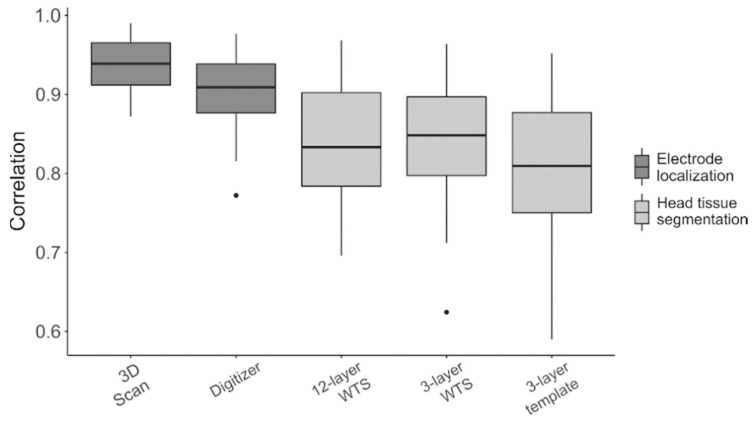
Correlation between the reference connectivity matrices and those obtained when introducing errors in the head model. These errors were related to the electrode localization technique (dark grey) or to the head tissue segmentation method (light grey). Black dots represent the outliers.

When comparing the two factors contributing to head model inaccuracy, i.e., the electrode positioning technique and the head tissue segmentation method, we observed that the former one had significantly less impact on the estimation of the connectivity matrices (Figure 7). Indeed, the z-values for 3D scanning and digitizer techniques were always significantly higher (*p* < 0.001) than those obtained for the different head tissue segmentation methods (Table S4).

**Figure 8:**
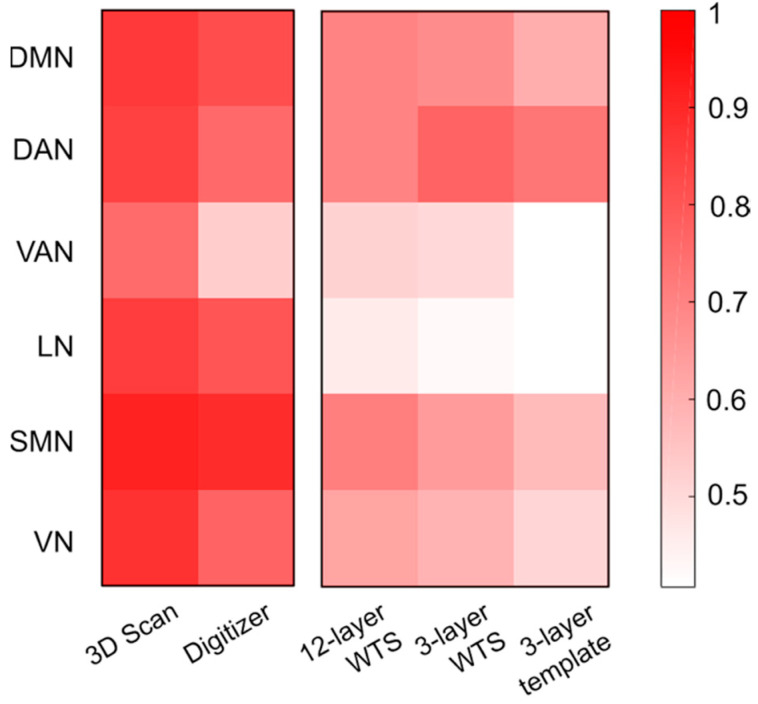
Impact of head modeling on the intra- and inter-network connectivity differences, for each RSN. This was assessed by calculating the correlation with values obtained using the reference analysis workflow. Two electrode localization techniques (*3D Scan* and *Digitizer*—on the **left**) and three head tissue segmentation methods (*12-layer WTS*, *3-layer WTS* and *3-layer template*—on the **right**) were tested.

Lastly, we contrasted intra- and inter-network connectivity values for each resting-state network and frequency band, and examined the impact of head model inaccuracy (Figure S5). A correlation analysis conducted using the standard analysis workflow as a reference confirmed that the head tissue segmentation had a stronger impact on connectivity estimation than electrode positioning (Figure 8 and Figure S6). In line with other analyses conducted in this study (Figures S3 and S4), VAN and LN were found to be the most sensitive networks to variations in the head modeling strategy, whereas DAN was the most robust one.

## 4. Discussion

In recent years, an increasing number of studies have performed source-level functional connectivity analyses using hdEEG [1,10,18,19,49,68,69]. The reliability of the source activity reconstruction from hdEEG data, which is needed for estimating functional interactions between brain regions, does not depend solely on the density and coverage of the montage, but also on the workflow used for data analysis [14,16,17,27,70]. The primary goal of this study was to specifically examine the robustness of EEG-RSN reconstruction using different electrode localization and head tissue segmentation approaches.

### 4.1. Impact of Electrode Localization Error

The precision of electrode localization can be generally ascribed to two main factors: the single-electrode localization and the overall co-registration of the sensor positions over the head shape [48]. The results of our analyses show that random errors in electrode positioning (i.e., single-electrode localization errors) had generally a larger impact than the systematic errors (i.e., sensor co-registration errors) on the EEG connectivity results (Figure 6). The effect of random and systematic errors was significantly different for average displacements equal to 0.5 cm, with the former one clearly hindering the reconstruction of EEG connectivity patterns. For greater error magnitudes, instead, the effects of the two error types were more comparable, although systematic errors always had a more limited impact on EEG connectivity measures than random errors. Notably, for displacements as low as 0.25 cm there was no significant difference between the random and systematic errors, and relatively high correlations with our benchmark results. This finding is in line with previous literature, suggesting that electrode positioning errors smaller than 0.5 cm are negligible for an adequate brain source reconstruction [71,72].

When collecting electrode locations to be included in the individual head model, commonly-used electrode positioning techniques can be affected by both random and systematic errors, but their respective magnitude can vary consistently [41,42,45,46,48,65,73]. In this study, we modeled electrode positioning errors of the 3D scanning, assuming that the related measurements were affected by small systematic errors (0.25 mm) and that random errors were negligible. We also modeled electrode positioning errors of the digitizing technique as prone to both error types. The results of our study clearly show that the different measurement accuracy of these two techniques [44,45,48] resulted in evident differences in the EEG-RSN connectivity results (Figure 7 and Table S4).

### 4.2. Impact of Head Tissue Segmentation

The accuracy of the tissue segmentation can be related to the space in which the segmentation is performed (individual, as in MR-TIM, or template, as in WTS) and to the number of considered tissues. Our results evidence that warping a template segmentation on the individual MR image (*12-* or *3-layer WTS*) [10] was a substantially less accurate approach than performing head tissue segmentation directly on the individual MR image itself [25] (Figure 7 and Table S4). We expected an improvement in performance when discriminating twelve tissue compartments (*12-layer WTS*) instead of only three (*3-layer WTS*). However, our tests did not reveal any significant difference when fewer tissues were included in the head model. This result may simply suggest that the WTS technique may not be sufficiently accurate under several conditions, and that it would be preferable to use MR-TIM for head tissue segmentation [25].

### 4.3. Differential Impact of Electrode Localization and Head Tissue Segmentation

As already mentioned, the head model used for reconstructing cortical activity combines information about electrode locations and head tissue distribution. In our study, we aimed to test which of these two factors has a stronger impact on EEG connectivity results. Our simulations yielded significantly higher correlations with the reference connectivity values when using different electrode positioning techniques (*3D Scan* and *Digitizer*), rather than when considering different head tissue segmentation methods (*12-* or *3-layer WTS*) (Figure 7 and Table S4). In particular, suboptimal performance was achieved when using the template head model (*3-layer template*), created from the electrode positions and the template MR image defined in MNI space. This finding highlights the importance of including subject-specific information in the head model, namely real electrode positions and individual tissue segmentation [27,28,29].

### 4.4. Analysis of Robustness for Different Frequency Bands and Networks

The use of hdEEG has recently enabled the investigation of frequency-dependent functional interactions of several cortical regions and networks [10,18,49]. The results of our study clearly reveal that variations in EEG connectivity due to head model errors depend on the network and the frequency band considered (Figure 5 and Figures S2–S5). Specifically, the alpha band was the most robust in regard to head model variations, whereas the delta band was the most sensitive one (Figures S3 and S4). It should be noted that we analyzed the electrophysiological activity of participants in the resting state, a condition in which the alpha rhythm dominates the interactions among all brain regions [11,74]. Therefore, the robustness of the functional connectivity patterns in each band could vary according to the specific experimental conditions. In particular, the strongest brain oscillations are expected to be the least influenced by changes in the head model. We also found that DMN, DAN and SMN were relatively robust, whereas VAN and LN had large connectivity variations in the different test conditions (Figure 8 and Figures S3, S4 and S6). It should be considered that the number and the spatial distribution of network nodes over the cortex may influence the robustness of RSN reconstruction: VAN and LN are strongly lateralized networks with main nodes in the same hemisphere, whereas the other networks have core regions on both hemispheres. The robustness of the EEG-RSN reconstruction may also be influenced by other factors related to the RSN spatial distribution: DMN and DAN encompass deeper brain regions surrounded by uniformly distributed tissue interfaces, whereas key regions of VAN and LN are located closer to air–tissue interfaces, which are more likely to cause distortions in the electrical fields and therefore to affect the accuracy of the source reconstruction.

### 4.5. Study Limitations

A number of limitations in this study must be considered. First, we acknowledge the fact that we did not have the possibility to locate the EEG electrodes directly from the MR image, which would have assured error-free localization [73,75]. To minimize the effect of any electrode positioning error associated with the GPS technique, we projected the obtained electrode coordinates over the head shape [42,73] and manually corrected any apparent imprecision in electrode positioning. Additionally, we did not collect real electrode positions with several devices to compare our reference with; we therefore simulated positioning errors for 3D scanning and digitizing techniques, respectively, using error type and magnitude reported in previous studies. Similarly, a manually segmented MR image was not available to be used in our study. The analysis workflow that we used as a benchmark included MR-TIM, which was proven to outperform other commonly-used head tissue segmentation techniques [25]. We would also like to mention that resting-state EEG recordings used in this study were collected for 5 min in each participant [10,16], and it would be important to test if EEG-RSN reconstruction is stable for different EEG recording lengths. Furthermore, EEG connectivity results were based on time–frequency analyses conducted using short-time Fourier transform with uniform window length for all frequency bands, as in our previous studies [10,12,49]. The Wavelet transform could be alternatively used, such that the window length is adaptively defined based on the frequency; this may increase the sensitivity of the connectivity analyses for lower-frequency bands. Lastly, we assessed the impact of different head models on EEG connectivity by calculating the similarity of EEG connectivity results with respect to a standard configuration. This solution permitted us to conduct only relative comparisons among our test datasets; to the best of our knowledge, an absolute threshold level against which assessing the correspondence of two connectivity profiles can hardly be defined.

## 5. Conclusions

Network connectivity estimation from hdEEG data can be performed using an electrophysiological source imaging workflow that includes signal pre-processing, head model creation and brain activity reconstruction at the cortical level. In this study, we characterized to what extent head model variations associated with electrode positioning and head tissue segmentation influence EEG connectivity patterns. Our results suggest that inaccuracies in tissue segmentation have a stronger impact than those related to the electrode localization. Additionally, we observed that strongly lateralized networks, such as VAN and LN, were substantially less robust than bilateral networks. We hope that the present study may provide important information for researchers planning new EEG studies, supporting their choice of a suitable electrode positioning and head tissue segmentation approach, respectively. We suggest that future studies could perform test–retest analyses, which offer an alternative way to estimate the reliability of EEG connectivity results with respect to different head modeling approaches. Finally, it may be interesting to extend our findings by testing the impact of head modeling on EEG connectivity using other datasets, collected, for instance, in healthy older adults or in neurological patients.

## Figures and Tables

**Table 1:** ANOVA testing for electrode localization errors. Four factors were assessed: resting-state network (DMN, DAN, VAN, LN, SMN and VN), frequency band (delta; theta; alpha; beta; gamma), error magnitude (0.25; 0.5; 0.75; 1 cm) and error type (systematic; random).

Electrode Localization Errors	df	Sum Squares	Mean Square	F	*p*-Value
Network	5	6.58	1.32	79.39	<0.001
Band	4	18.10	4.53	273.19	<0.001
Error magnitude	3	7.28	2.43	146.54	<0.001
Error type	1	0.46	0.46	27.72	<0.001
Residuals	226	3.74	0.02		
Total	239	36.16			

**Table 2:** ANOVA testing for head tissue segmentation methods. Three factors were assessed: resting-state network (DMN; DAN; VAN; LN; SMN; VN), frequency band (delta; theta; alpha; beta; gamma) and segmentation method (*12-layer WTS*; *3-layer WTS*; *3-layer template*).

Head Tissue Segmentation Methods	df	Sum Squares	Mean Square	F	*p*-Value
Network	5	1.18	0.24	19.52	<0.001
Band	4	5.83	1.46	120.42	<0.001
Segmentation method	2	0.29	0.15	12.17	<0.001
Residuals	78	0.94	0.01		
Total	89	8.24			

## Data Availability

Data are available from the corresponding author (D.M.) upon request.

## Supplementary Materials

The following are available online at https://www.mdpi.com/article/10.3390/brainsci11060741/s1, Table S1: Definition of the 21 seeds used for analysis; Table S2: Definition of the simulated electrode localization errors due to different positioning techniques; Table S3: Correspondence between the reference connectivity matrices and those obtained when introducing electrode localization errors; Table S4: Comparative analysis of the z-values expressing the correspondence between the reference connectivity matrices and the matrices obtained when introducing errors in the head model; Figure S1: Representation of the head tissues in template space; Figure S2: Comparison of intra- and inter-network connectivity values for each frequency band and network; Figure S3: Estimated marginal means plots for each of the four factors included in the ANOVA test for electrode localization errors; Figure S4: Estimated marginal means plots for each of the three factors included in the ANOVA test for the data processed with different head tissue segmentation methods; Figure S5: Contrasts between intra- and inter-network connectivity, for different head models; Figure S6: Scatterplots of intra- and inter-network connectivity differences for different head models, compared to the corresponding values obtained using the reference analysis workflow.
