# Supplementary material for: Detection of Resting-State Functional Connectivity from High-Density Electroencephalography Data: Impact of Head Modeling Strategies
Source: Brain Sci. 2021 Jun 3;11(6):741. doi: 10.3390/brainsci11060741 (PMC8226780; doi:10.3390/brainsci11060741)
Supplement: Supplementary file 1 [file brainsci-11-00741-s001.zip › brainsci-1187586-supplementary.pdf]

Supplementary Materials

Detection of resting-state functional connectivity from high-density electroencephalography data: impact of head modelling strategies

Gaia Amaranta Taberna, Jessica Samogin, Marco Marino and Dante Mantini

Table S1. For each network, the selected seeds, their full names, abbreviations and coordinates in MNI space are indicated.

Network	*Seed name*	*Abbreviation*	*MNI coordinates*
Default Mode Network (DMN)	Posterior cingulate cortex	lANG	[-32,-76,44]
	Medial prefrontal cortex	rANG	[57,-63,17]
	Left angular gyrus	PCC	[-2,-50,30]
	Right angular gyrus	MPFC	[-2,32,-10]
Dorsal Attention Network (DAN)	Left Frontal Eye Field	lIPS	[-34,-48,44]
	Right Frontal Eye Field	rIPS	[33,-57,41]
	Left Inferior Parietal Sulcus	lFEF	[-32,-6,49]
	Right Inferior Parietal Sulcus	rFEF	[36,-19,44]
Ventral Attention Network (VAN)	Right Temporo-Parietal Junction	rTPJ	[60,-43,16]
	Right Inferior Frontal Gyrus	rIFG	[45,29,10]
Language Network (LN)	Left Temporo-Parietal Junction	lTPJ	[-54,-33,-4]
	Left Inferior Frontal Gyrus	lIFG	[-47,14,1]
Somatomotor Network (SMN)	Supplementary Motor Area	SMA	[-1,-17,55]
	Left Primary Somatosensory Cortex	lS1	[-45,-17,49]
	Right Primary Somatosensory Cortex	rS1	[45,-17,49]
	Left Secondary Somatosensory Cortex	lS2	[-42,-13,10]
	Right Secondary Somatosensory Cortex	rS2	[42,-13,10]
Visual Network (VN)	Left human ventral Visual 4 area	lV4v	[-27,-81,-13]
	Right human ventral Visual 4 area	rV4v	[24,-82,-16]
	Left Visual 5 area	lV5	[-44,-67,1]
	Right Visual 5 area	rV5	[44,-73,-1]

Table S2. Electrode localization errors considered for *3D Scan* and *Digitizer* techniques, respectively.

ELECTRODE LOCALIZATION ERRORS (cm)	*Systematic Error*	*Random Error*
3D Scan	0.25	0
Digitizer	0.5	0.25

Table S3. Correspondence between the reference connectivity matrices and those obtained when introducing electrode localization errors. A two-tailed Wilcoxon signed rank paired test was performed between values obtained for systematic and random errors, respectively, for each error magnitude.

ERROR MAGNITUDE (cm)	*z-values*	*p-values*
0.25	–0.237	0.813
0.5	4.762	< 0.001
0.75	2.026	0.043
1	2.705	0.007

Table S4. Comparative analysis of the z-values expressing the correspondence between the reference connectivity matrices and the matrices obtained when introducing errors in the head model. Different electrode localization techniques (*3D Scan* and *Digitizer*) and different head tissue segmentation methods (*12-layer WTS, 3-layer WTS, 3-layer template*) were assessed. A two-tailed Wilcoxon signed rank paired test was used to perform comparisons between models. Probabilities were corrected for multiple comparisons using the Bonferroni method.

ADJUSTED VALUES*		*Digitizer*	*12-layer WTS*	*3-layer WTS*	*3-layer template*
3D Scan	z-value<br>p-value	4.55<br>< 0.001	4.78<br>< 0.001	4.78<br>< 0.001	4.78<br>< 0.001
Digitizer	z-value<br>p-value	/	4.72<br>< 0.001	4.78<br>< 0.001	4.78<br>< 0.001
12-layer WTS	z-value<br>p-value	/	/	–0.50<br>ns	3.67<br>0.002
3-layer WTS	z-value<br>p-value	/	/	/	4.54<br>< 0.001

* Bonferroni correction

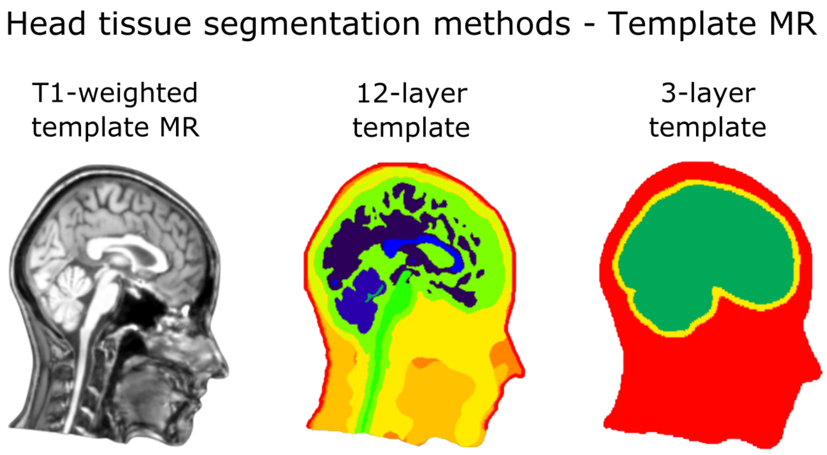

Figure S1. Representation of the head tissues in template space. From left to right: T1-weighted template MR image; 12-layer segmentation; 3-layer segmentation.

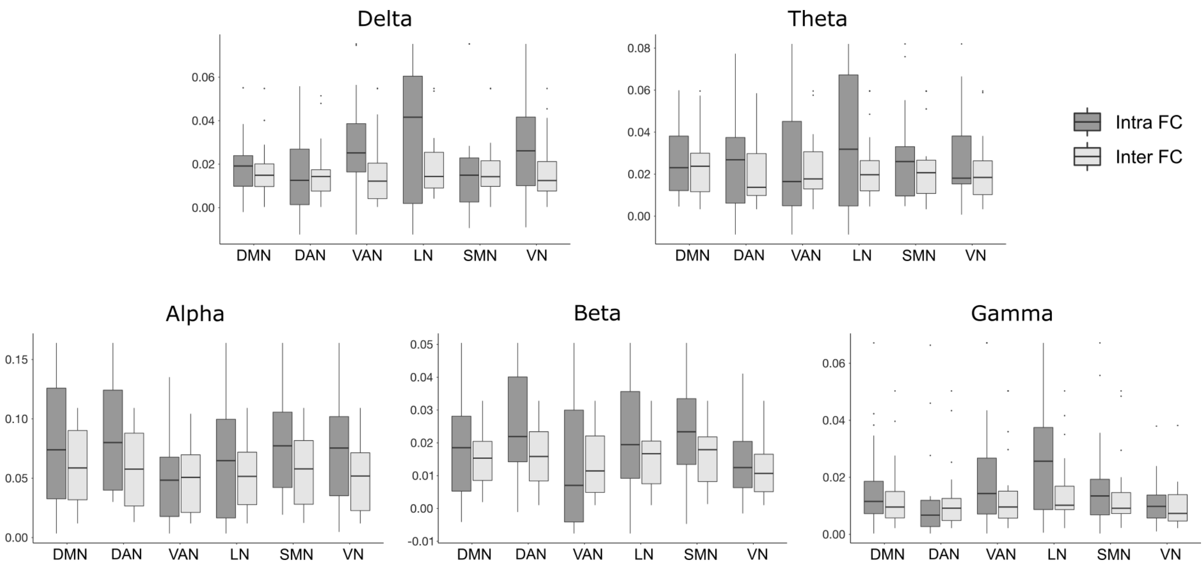

Figure S2. Comparison of intra- (dark grey) and inter- network (light grey) connectivity values for each frequency band and network.

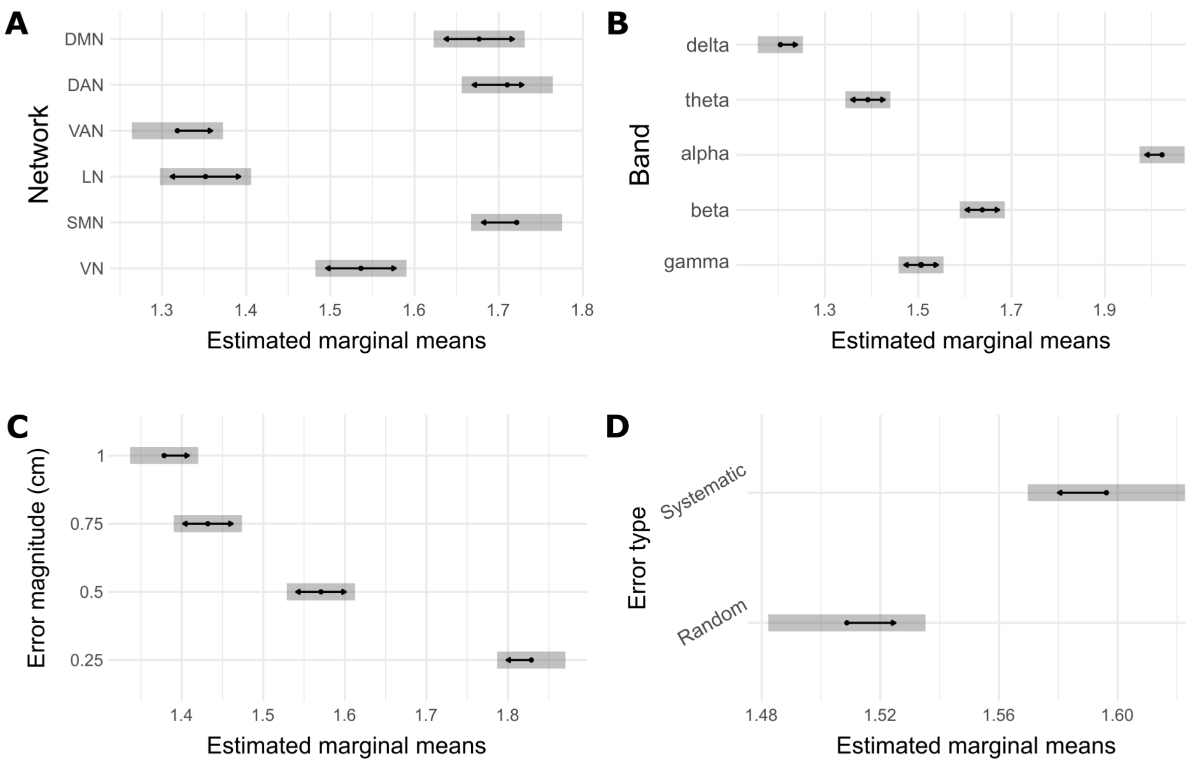

Figure S3. Estimated marginal means plots for each of the four factors included in the ANOVA test for electrode localization errors: (A) resting state network, (B) frequency band, (C) error magnitude and (D) error type. The black dots and the grey rectangles show the estimated means and their 95% confidence intervals, respectively.

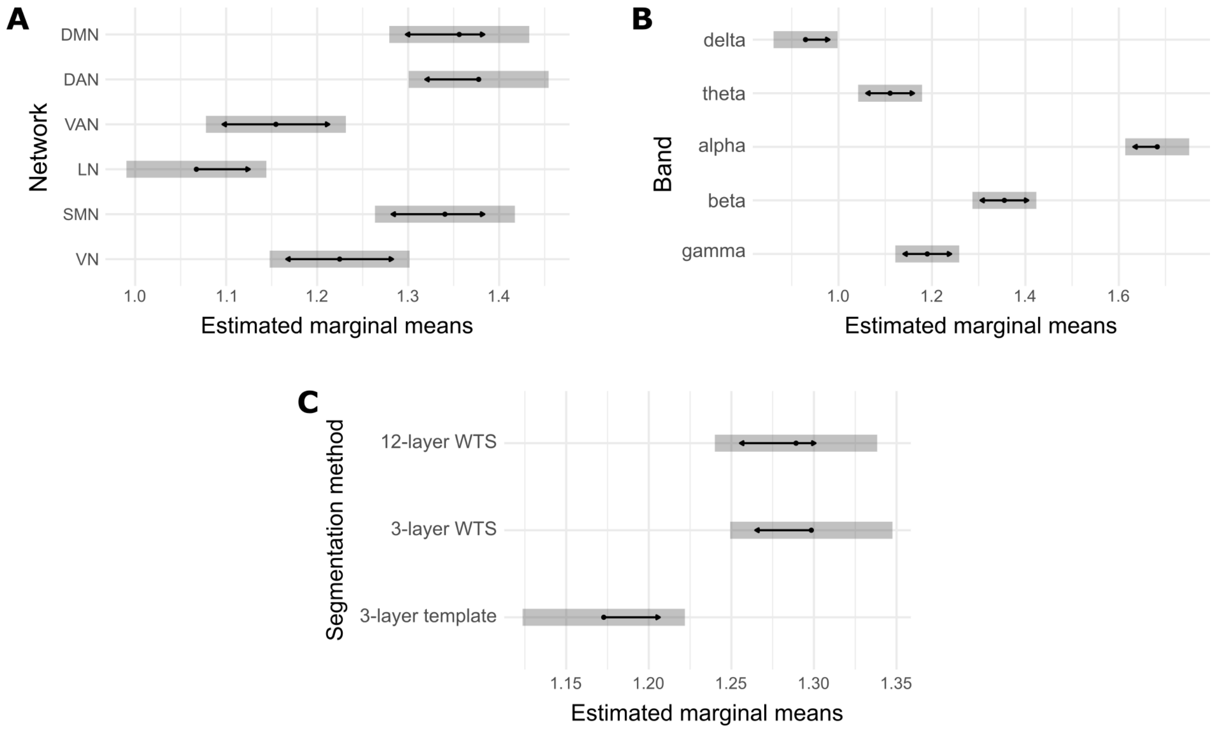

Figure S4. Estimated marginal means plots for each of the three factors included in the ANOVA test for the data processed with different head tissue segmentation methods: (A) resting state network, (B) frequency band and (C) segmentation method. The black dots and the grey rectangles show the estimated means and their 95% confidence intervals, respectively.

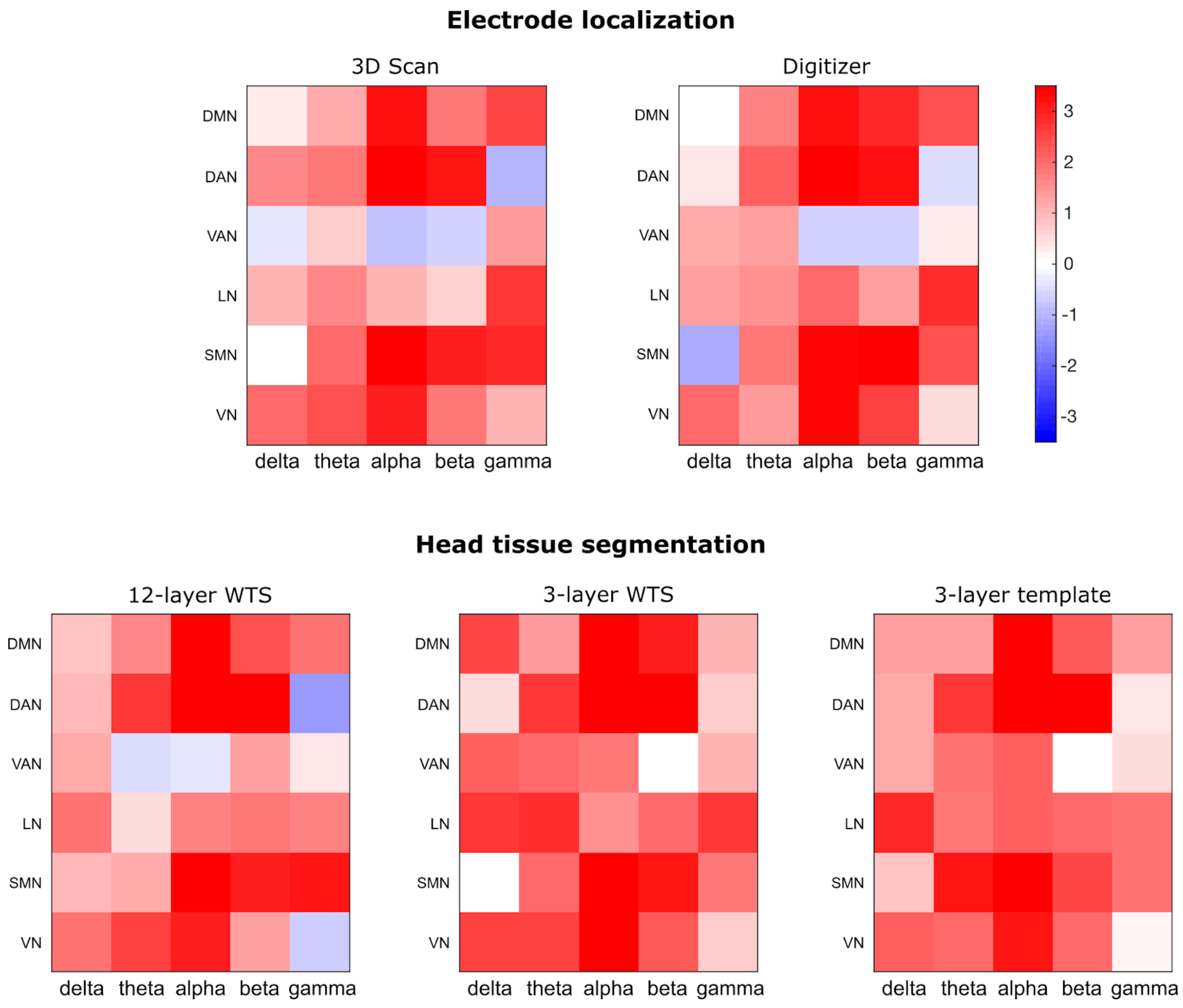

Figure S5. Contrasts between intra- and inter-network connectivity, for different head models. The z-values of the Wilcoxon signed rank test between intra- and inter-network connectivity are represented. The top and bottom row show the matrices obtained with different electrode localization techniques (*3D Scan* and *Digitizer*) and head tissue segmentation methods (*12-layer WTS*, *3-layer WTS* and *3-layer template*), respectively.

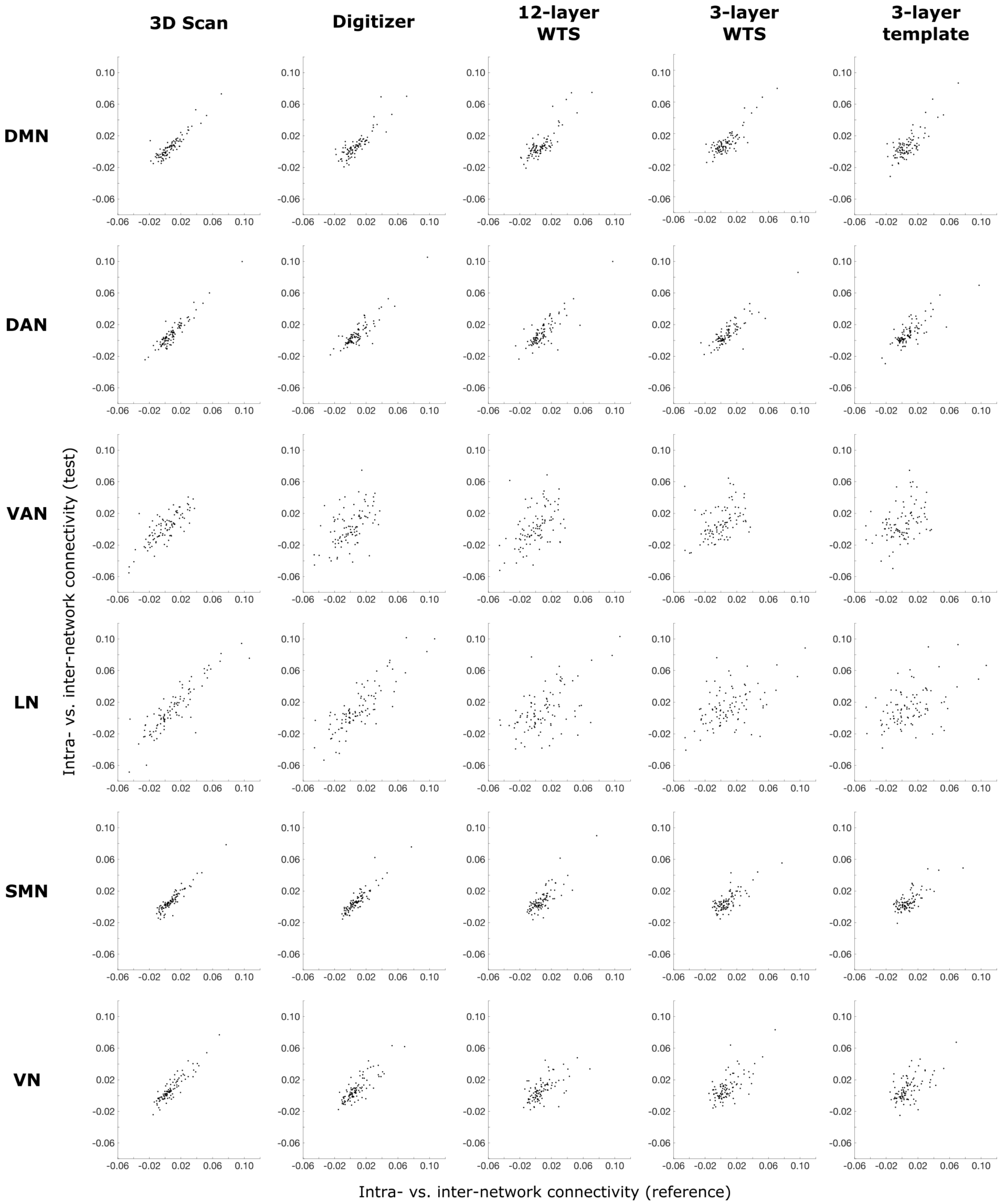

Figure S6. Scatterplots of intra- and inter-network connectivity differences for different head models, compared to the corresponding values obtained using the reference analysis workflow. Concerning the head models, two electrode localization techniques (*3D Scan* and *Digitizer* – fist two columns) and three head tissue segmentation methods (*12-layer WTS*, *3-layer WTS* and *3-layer template* – last three columns) were tested.
